# Dehydroepiandrosterone and Dehydroepiandrosterone Sulfate in Alzheimer's Disease: A Systematic Review and Meta-Analysis

**DOI:** 10.3389/fnagi.2019.00061

**Published:** 2019-03-29

**Authors:** Xiongfeng Pan, Xinyin Wu, Atipatsa C. Kaminga, Shi Wu Wen, Aizhong Liu

**Affiliations:** ^1^Department of Epidemiology and Health Statistics, Xiangya School of Public Health, Central South University, Changsha, China; ^2^Department of Mathematics and Statistics, Mzuzu University, Mzuzu, Malawi; ^3^Department of Obstetrics and Gynaecology, University of Ottawa, Ottawa, ON, Canada; ^4^Ottawa Hospital Research Institute, Ottawa, ON, Canada

**Keywords:** dehydroepiandrosterone, dehydroepiandrosterone sulfate, Alzheimer's disease, meta-analysis, systematic review

## Abstract

**Background and Purpose:** Previous studies found inconsistent results for the relationship between Alzheimer's disease and the levels of dehydroepiandrosterone and dehydroepiandrosterone sulfate. This study performed a systematic review and meta-analysis to evaluate previous studies' results on this relationship.

**Method:** Studies related to this outcome were obtained using a systematic search from the electronic databases of PubMed, Embase, Web of Science, and Psyc-ARTICLES in March 2018. The random-effects model was used to measure the strength of the association between Alzheimer's disease and the levels of dehydroepiandrosterone and dehydroepiandrosterone sulfate, using the standardized mean difference.

**Results:** Thirty-one eligible studies were included in the final analysis. There was no statistically significant association between the level of dehydroepiandrosterone and Alzheimer's disease (standardized mean difference: 0.51, 95% confidence interval: −0.44 to 1.45, *Z* = 1.06, *p* = 0.29). On the other hand, lower level dehydroepiandrosterone sulfate was observed in patients with Alzheimer's disease than in controls (standardized mean difference: −0.69, 95% confidence interval: −1.17 to −0.22, *Z* = −2.84, *p* < 0.01).

**Conclusion:** Decreased dehydroepiandrosterone sulfate concentrations may be an important indicator for Alzheimer's disease, although whether dehydroepiandrosterone sulfate could be used as a diagnostic tool requires further research.

## Introduction

Alzheimer's disease (AD) is a progressive neurodegenerative disorder with complex etiology characterized by intellectual decline including language breakdown, and memory loss (Scheltens et al., 2016). The incidence of AD has increased in recent years worldwide (The Lancet, 2016). Therefore, AD is a major public health problem worldwide. Early detection/diagnosis of AD may help to implement interventions, which may lead to better progress and reduced burden of the disease (Scheltens et al., 2016). There have been long preclinical phases before the full-blown dementia syndrome appears (Anderson et al., 2017). Biomarkers with high sensitivity and specificity can help in the investigation of the pathophysiology of AD and this may help detect AD as early as possible (Liu et al., 2018). Recent advances in cognitive neuroscience proposed that biomarkers such as dehydroepiandrosterone (DHEA) and dehydroepiandrosterone sulfate (DHEA-S) may be promising new options for the screening, treatment, and prevention of AD. In this regard, some studies have suggested that concentrations of DHEA and DHEA-S may decrease with age (Goncharov and Katsia, 2013). Physiologically, both DHEA and DHEA-S arise from the zona reticularis of the adrenal cortex (Rammouz et al., 2011a). In addition, certain neurons are also prone to producing small amounts of DHEA and DHEA-S. These substances (DHEA and DHEA-S) are abundant in the brain and regulate the activity of neurons through cell membrane receptors. Importantly, DHEA and DHEA-S are neuroactive steroids, which may contribute to the development of AD and play an important role in the brain aging by influencing synaptic connectivity and neuronal differentiation (Rammouz et al., 2011b).

The tendency of DHEA and DHEA-S to associate with AD brings many hypotheses. For example, the decreased DHEA and DHEA-S levels in AD individuals may have an important bearing in the pathogenesis of this disease in view of widely documented effects of DHEA and DHEA-S on memory facilitation, neuroprotection, neuronal plasticity and neurogenesis in different experimental models (Zdrojewicz and Ciszko, 2001; Klinge et al., 2018). In more concrete terms, the imbalance of DHEA and DHEA-S plays an important role in the progression of neurotransmission disarrangement, which would induce further the presence of extracellular deposits of Amyloid β (Aβ) protein, senile plaques, and intracellular fibrillary tangles, hence inducing symptoms of pre-dementia, like hypo-activity, gait disturbances and decline of cognitive functioning (Arbo et al., 2018). Interestingly, existing clinical research also suggest that AD patients show lower DHEA-S levels in striatum, cerebellum, and hypothalamus. Besides, a negative correlation was observed between DHEA-S and phospho-tau protein levels in hypothalamus, and between DHEA-S and Aβ peptide levels in striatum and cerebellum (Weill-Engerer et al., 2002; Jiménez-Rubio et al., 2017). In summary, DHEA-S has a very long half-life and hence may make an attractive biomarker by which to assess AD status or progression (Legrain et al., 2000).

Therefore, the preceding evidence supports the hypothesis that DHEA and DHEA-S are involved in the pathophysiology of cognitive decline symptoms in AD patients. However, results of other studies contradicted with this hypothesis. Therefore, there was need to conduct a meta-analysis, which can provide a gold-standard approach to data aggregation (Rasmuson et al., 2011). In addition, DHEA and DHEA-S have been marketed in many countries as health supplement (Racchi et al., 2003), without the rigorous scientific evaluation of its effect and potential risks. Thus, this study summarized results of previous studies on the association of DHEA and DHEA-S levels with AD and quantified the strength of this relationship.

## Methods

### Search Strategy and Selection Criteria

This systematic review and meta-analysis has been registered and the full protocol was uploaded to the International Prospective Register of Systematic Reviews website (CRD 42018112810). Moreover, this systematic review and meta-analysis followed the Cochrane Handbook guidelines, and reported results as recommended by the Preferred Reporting Items for Systematic Reviews, and Meta-Analyses (PRISMA) guidelines (Moher et al., 2010). We searched relevant studies/articles from four electronic databases: PubMed, Embase, Web of Science, and Psyc-ARTICLES. Our search was restricted to all articles published in English from inception of a database to March 8, 2018. An experienced librarian designed our search strategy, which involved keywords related to the following concepts: (1) dehydroepiandrosterone (DHEA) or dehydroepiandrosterone-sulfate (DHEA-S); and (2) Alzheimer's Disease (AD). The detailed search strategies for the four databases were listed in Appendix 1.

### Eligibility Criteria

Studies were considered eligible if they (1) were case control studies, which compared AD cases with healthy controls; (2) used AD diagnostic criteria based on standardized criteria, such as in the DSM or NINCDS-ARDRA. (3) measured DHEA and DHEA-S concentrations in AD patients and controls; (4) reported mean and standard deviation (SD) of DHEA and DHEA-S concentrations for AD patients, and controls. Studies were excluded if they (1) were review articles or case reports; (2) studied AD in combination with other mental illnesses; (3) Mild Cognitive Impairment (MCI) or vascular dementia patients, who used psychotropic medication or other medications which could influence the DHEA and DHEA-S levels; (4) were animal or *in vitro* experiments; (5) were gray literature (i.e., unpublished reports).

Two of the authors [XP and AC] independently screened articles and selected eligible studies. In case of disagreement the final decision was made in consultation with the corresponding author [AL].

### Data Extraction

After selecting the eligible studies, the two authors [XW and AC] independently extracted information on the following variables according to the purpose of this study: (1) name of the first author and publication year; (2) country of the study; (3) study design; (4) mean age and standard deviation (mean, SD) of subjects; (5) gender distribution of subjects; (6) AD assessment method; (7) DHEA and DHEA-S measurements, such as type of sample, sample collection time, storage temperatures, and assay methods; (8) mean and SD of DHEA and DHEA-S concentrations. All the extracted data were organized in EpiData 3.0 and saved in Excel.

### Quality Evaluation

The Newcastle-Ottawa Quality Assessment Scale (NOS) was used to assess the quality of the eligible studies (Wells et al., 2011). Each of them was evaluated based on three broad perspectives: (1) Selection (4 points): representativeness of the sample, adequacy of the definition and selection of controls; (2) Comparability (2 points): whether the subjects in different outcome groups are comparable based on the study design or analysis, and confounding factors are controlled; (3) Outcome (3 points): ascertainment of exposure, same method of ascertainment for cases, and controls, and non-response rate. According to the pre-specified scoring criteria of this instrument, the total scores ranged from 0 to 9 and studies scoring 7–9, 3–6, and 0–3 points were graded as high, moderate and low quality, respectively. This assessment was also done by the two authors [XW and AC] independently, and inconsistencies were resolved by a group discussion. The scoring method and scores for each aspect of the quality assessment are shown in the Appendix 2.

### Statistical Analysis

All analyses were carried out using R software (version R i386 3.4.2). First, we performed meta-analyses of all the enrolled studies to compare concentrations of DHEA and DHEA-S between AD patients and healthy controls. The means and corresponding standard deviations (SDs) for DHEA concentrations were converted to nmol/mL, whereas for DHEA-S they were converted to μmol/mL. Then the standardized mean differences (SMD) with corresponding 95% confidence intervals (CIs) were calculated to evaluate the strength of the relationship between AD and the concentrations DHEA and DHEA-S (Higgins et al., 2003; Pan et al., 2018a). The effect size was considered large when SMD was >0.8, moderate when it was between 0.5 and 0.8, and low when it was lower than 0.5. Heterogeneity was assessed by the Cochran's Q-statistic, the *I*^2^ test was also used to reflect the possibility of heterogeneity between enrolled studies (Higgins et al., 2003; Pan et al., 2018b). The *I*^2^ = 0% indicated no heterogeneity, and the *I*^2^ = 100% indicated maximal heterogeneity. In order to supply quantitative evidence from all selected studies and minimize the variance of the summary SMD, we used random-effects model (when *I*^2^> 50%) or fixed-effects model (when *I*^2^≤ 50%) (Berkey et al., 1995).

Subgroup analyses were conducted to explore the impact of type of tissue samples used, sample storage temperature, and assay method used for the measurement of DHEA and DHEA-S, country of study, NOS of studies, age of AD patients, and AD assessment tool used. Sensitivity analysis was performed to examine whether results could be influenced significantly by excluding individual studies in turn. Finally, the Egger funnel plot was constructed to assess its symmetry for the presence of any publication bias, and this was verified using Egger's linear regression test (Egger et al., 1997). In all the statistical tests, the level of significance for the effect size estimation was set at the 5%, and all tests were two-sided.

## Results

### Literature Search

**Figure 1:**
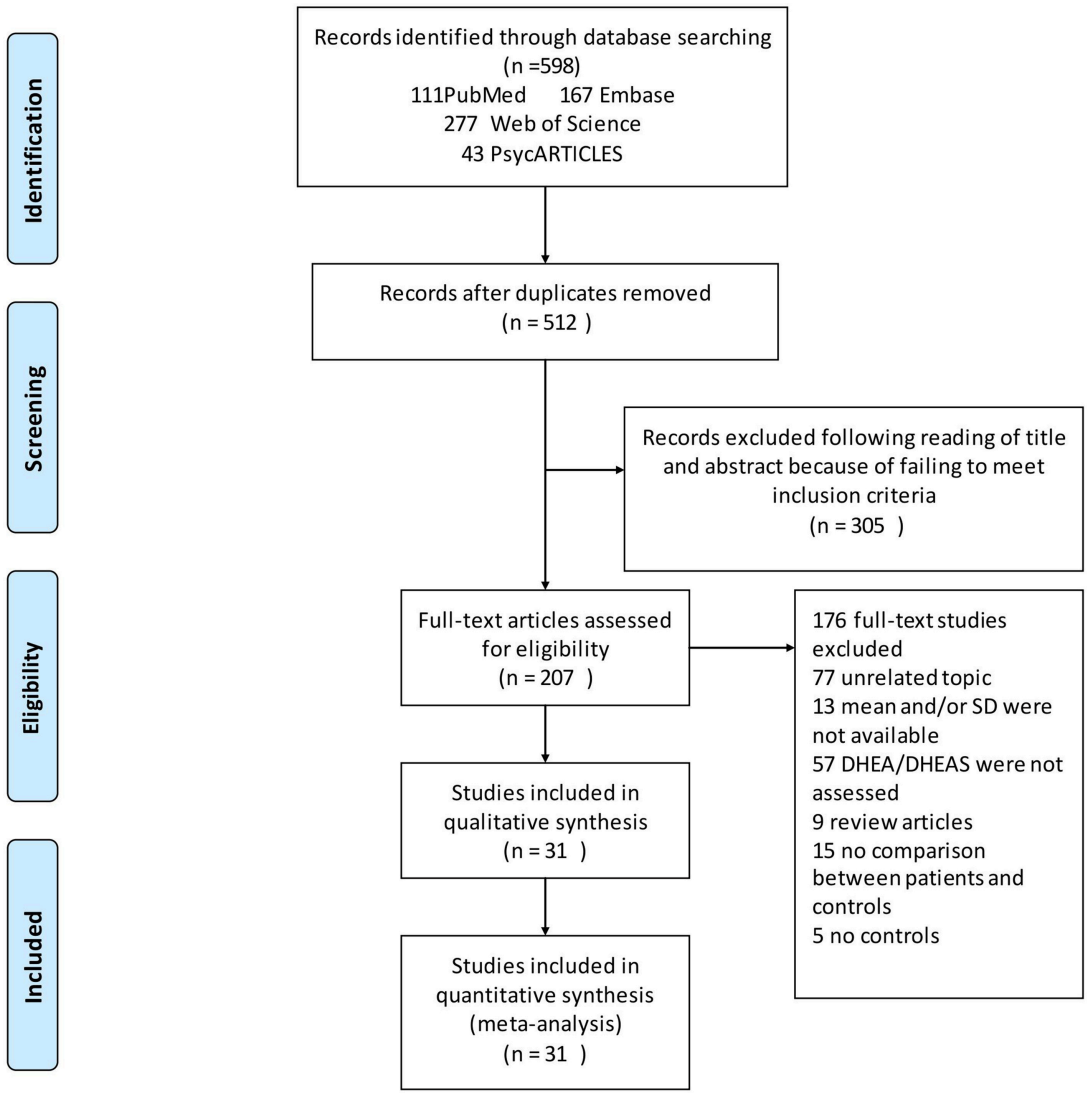
Flow chart of study selection. Showing the process by which relevant studies retrieved from the databases were assessed and selected or excluded.

The search strategies yielded 598 relevant articles, of which, 111 were from PubMed, 167 from Embase, 277 from Web of Science, and 43 from PsycARTICLES. A total of 31 articles met the inclusion criteria and were included in the final analysis (Figure 1).

### Characteristics of Eligible Studies

Table 1 presents the characteristics of the 31 eligible studies. Altogether, these studies compared DHEA-S concentrations between 1,017 AD patients and 1,171 healthy controls, and DHEA concentrations between 318 AD patients and 297 healthy controls. The NOS scores of these studies varied between 5 and 8, with 4 studies graded as high quality and 27 as moderate quality.

**Table 1:** Characteristics of studies included in the meta-analysis.

**Study**	**Material**	**Design**	**Country**	**NOS**	**Female**	**Mean Age**	**AD Assessment**	**Collection time**	**Methods**	**Frozen**	**Severity**
Aldred and Mecocci, 2010	Plasma	Case-control	UK	5	45 (63%)	80.0 ± 4.0	NINCDS-ADRDA	NR	ELISA	−80°C	Moderate
Armanini et al., 2003	Plasma	Case-control	Italy	5	18 (78%)	68.0 ± 4.3	NINCDS-ADRDA	AM	ELISA	NR	Moderate
Attal-Khémis et al., 1998	Serum	Case-control	France	6	10 (100%)	82.1 ± 7.0	NINCDS-ADRDA	AM	GC/MS	−80°C	Severe
Bo et al., 2006	Serum	Case-control	Italy	8	112 (71%)	75.5 ± 6.7	NINCDS-ARDRA	NR	ELISA	NR	Moderate
Brown et al., 2003	CSF	Case-control	USA	5	6 (50%)	74.6 ± 7.2	DSM	NR	RIA	−80°C	Severe
Carlson et al., 1999	Plasma	Case-control	Canada	7	26 (50%)	75.9 ± 1.1	NINCDS-ARDRA	AM	RIA	−50°C	Mild
Cho et al., 2006	Plasma	Case-control	Korea	6	20 (100%)	75.2 ± 7.7	NINCDS-ARDRA	AM	HPLC	−20°C	Mild
Ferrari et al., 2000	Serum	Case-control	Italy	5	NR	81.4 ± 0.8	NINCDS-ARDRA	AM	RIA	−20°C	Severe
Gulnora et al., 2015	Serum	Case-control	Uzbekistan	6	18 (30%)	61.3 ± 5.7	NINCDS-ARDRA	NR	ELISA	NR	Moderate
Hillen et al., 2000	Plasma	Case-control	Germany	8	14 (50%)	87.2 ± 1.9	NINCDS-ARDRA	AM	ELISA	NR	Moderate
Hoskin et al., 2004	Serum	Case-control	USA	6	179 (100%)	80.7 ± 7.3	NINCDS-ARDRA	NR	RIA	NR	Moderate
Leblhuber et al., 1991	Serum	Case-control	Austria	6	10 (100%)	75.4 ± 4.6	DSM-III	AM	ELISA	−20°C	Severe
Leblhuber et al., 1992	Plasma	Case-control	Austria	5	10 (56%)	46.2+21.2	DSM-III	AM	ELISA	−20°C	Moderate
Leblhuber et al., 1993	Plasma	Case-control	Austria	6	11 (54%)	76.3 ± 7.1	DSM-III	AM	ELISA	−20°C	Severe
Magri et al., 2000	Serum	Case-control	Italy	5	14 (61%)	80.5 ± 2.5	NINCDS-ARDRA	AM	RIA	−20°C	Moderate
Marx et al., 2006	CSF	Case-control	USA	5	0 (0%)	83.0 ± 7.5	NINCDS-ARDRA	NR	GC/MS	NR	Severe
Masera et al., 2002	Serum	Case-control	Italy	6	6 (38%)	66.9 ± 1.9	NINCDS-ARDRA	AM	RIA	NR	Moderate
Murialdo et al., 2000	Plasma	Case-control	Italy	6	11 (79%)	72.1 ± 6.7	NINCDS-ARDRA	AM	RIA	−20°C	Moderate
Naylor et al., 2007	CSF	Case-control	USA	5	NR	81.0 ± 6.5	DSM	NR	GC/MS	NR	Severe
Naylor et al., 2010	CSF	Case-control	USA	5	23 (58%)	82.0 ± 3.7	NINCDS-ARDRA	NR	GC/MS	NR	Severe
Näsman et al., 1991	Serum	Case-control	Sweden	5	51 (59%)	77.6 ± 0.7	DSM-III	AM	RIA	NR	Severe
Nasman et al., 1995	Serum	Case-control	Sweden	6	10 (18%)	74.6 ± 6.5	NINCDS-ARDRA	AM	RIA	−70°C	Moderate
Näsman et al., 1996	Plasma	Case-control	Sweden	8	15 (65%)	74.2 ± 7.4	NINCDS-ARDRA	AM	RIA	−70°C	Moderate
Rasmuson et al., 1998	Serum	Case-control	Sweden	6	8 (62%)	78.0 ± 8.4	NINCDS-ARDRA	AM	RIA	NR	Moderate
Rasmuson et al., 2002	Serum	Case-control	Sweden	6	21 (64%)	76.4 ± 7.8	NINCDS-ARDRA	AM	RIA	4°C	Moderate
Ray et al., 2013	Serum	Case-control	India	5	12 (30%)	68.5 ± 3.9	DSM-IV	NR	ELISA	−20°C	Severe
Schneider et al., 1992	Plasma	Case-control	USA	5	20 (57%)	69.3 ± 6.9	DSM-III	AM	RIA	NR	Moderate
Schupf et al., 2006	Serum	Case-control	USA	5	17 (100%)	55.2 ± 1.5	NINCDS-ARDRA	AM	RIA	−20°C	Mild
Solerte et al., 1999	Serum	Case-control	Italy	6	8 (57%)	76.0 ± 6.1	DSM-III	AM	RIA	NR	Moderate
Sunderland et al., 1989	Plasma	Case-control	USA	5	6 (60%)	61.4 ± 7.9	DSM	NR	RIA	−70°C	Mild
Yanase et al., 1996	Serum	Case-control	Japan	5	12 (63%)	74.7 ± 6.7	DSM-III	NR	RIA	−20°C	Moderate

*USA, United States of America; UK, the United Kingdom; AD, Alzheimer's disease; RIA, radioimmunoassay; CSF, cerebrospinal fluid; ELISA, enzyme linked immunosorbent assay; HPLC, High performance liquid chromatography-tandem mass spectrometry; GC/MS, Gas Chromatography-Mass Spectrometer; NR, not report; DSM, Diagnostic, and Statistical Manual of Mental Disorders; NOS, Newcastle-Ottawa Quality Assessment Scale*.

### Overall Comparison

**Figure 2:**
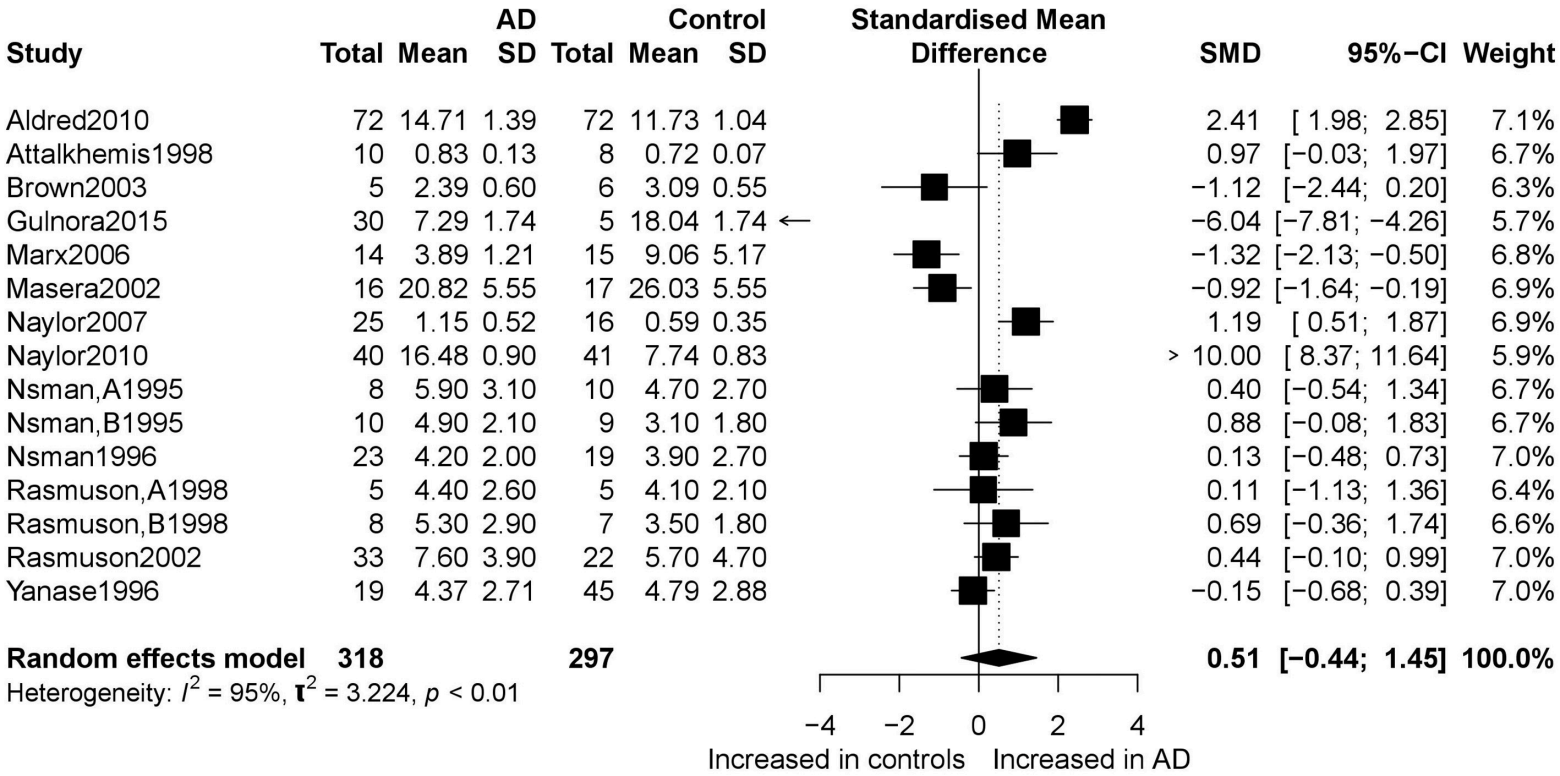
Forest plot of DHEA between AD participants and controls. Study effect sizes of DHEA differences between Alzheimer's disease and controls. Each data marker represents a study, and the size of the data marker is proportional to the total number of individuals in that study. The summary effect size for each DHEA is denoted by a diamond. AD, Alzheimer's disease; SMD, standardized mean difference.

**Figure 3:**
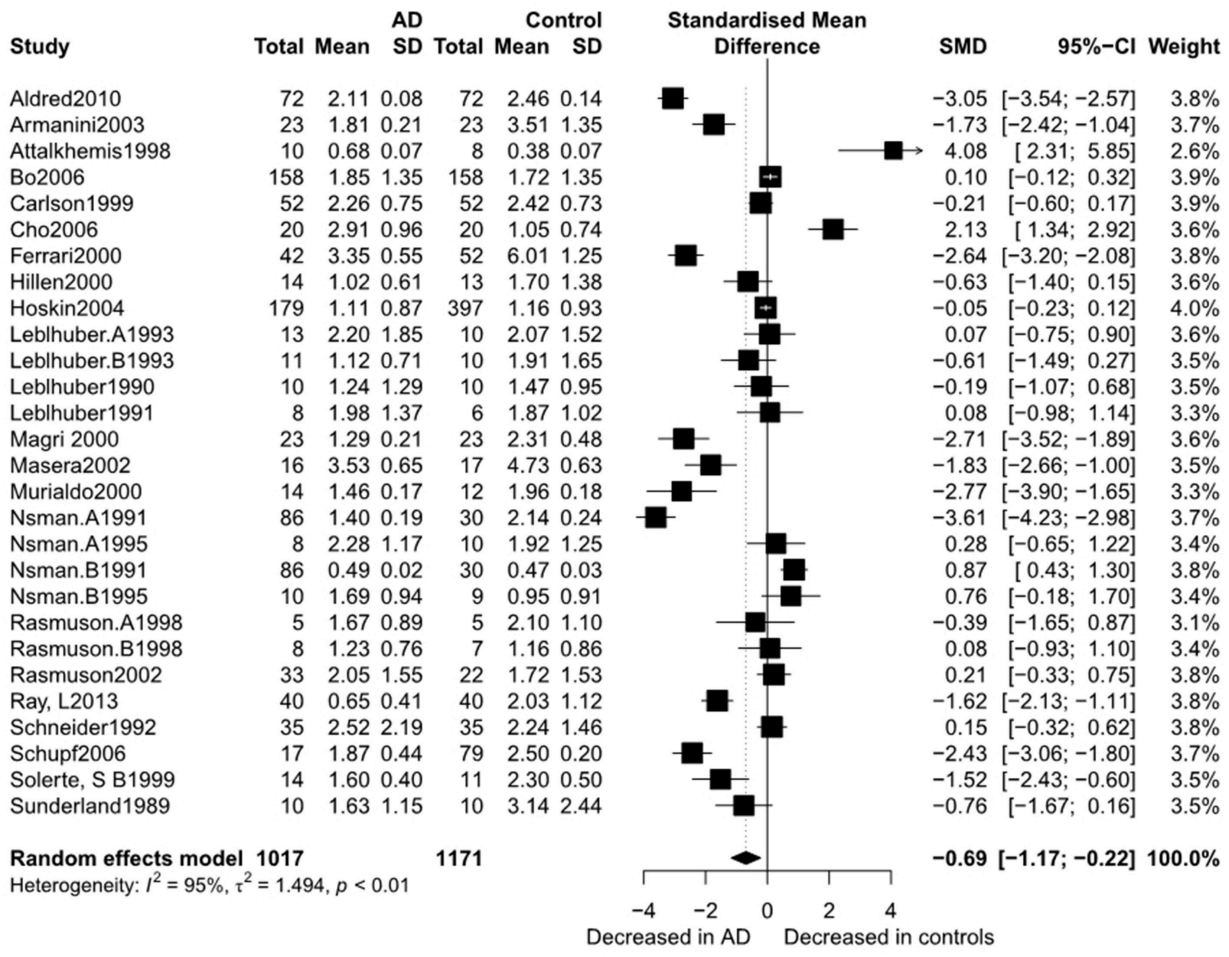
Forest plot of DHEA-S between AD participants and controls. Study effect sizes of DHEA-S differences between Alzheimer's disease and controls. Each data marker represents a study and the size of the data marker is proportional to the total number of individuals in that study. The summary effect size for each DHEA-S is denoted by a diamond. AD, Alzheimer's disease; SMD, standardized mean difference.

Figure 2 presents the results of the meta-analysis using random-effects model. Taking the 15 studies which compared DHEA concentrations between AD patients and healthy controls, the results showed that there was no significant difference (k = 15, SMD = 0.51, 95%CI: −0.44 to 1.45, *Z* = 1.06, *p* = 0.291), and heterogeneity was considerable (*I*^2^= 95.4%). A similar analysis on 28 studies comparing DHEA-S concentrations between 1,017 AD patients and 1,171 healthy controls showed that DHEA-S levels were significantly lower in AD patients than in the controls (k = 28, SMD = −0.69, 95% CI: −1.17 to −0.22, *Z* = −2.84, *p* = 0.004), but with high heterogeneity (*I*^2^ = 95.3%). Figure 3 shows the forest plot of DHEA-S concentrations for both groups.

### Subgroup Analyses

Tables 2, 3 show the results of subgroup analyses. Higher DHEA concentrations were observed in AD patients than in healthy controls (*k* = 5, SMD = 2.54, 95% CI: 0.32 to 4.76, *Z* = 2.24, *p* = 0.025) for studies with patients aged 80 years or older. Also, DHEA concentrations were significantly higher in AD patients than in healthy controls (k = 6, SMD = 0.40, 95%CI: 0.08 to 0.72, *Z* = 2.46, *p* = 0.014) with no heterogeneity (*I*^2^= 0%), when studies from Sweden only were considered. Furthermore, heterogeneity was considerably lower when morning samples or Radioimmunoassay (RIA) was used in the assay of DHEA. On the other hand, no significant difference on DHEA-S concentrations was found between AD patients and healthy controls, when a plasma or assay method other than RIA was used. Additionally, no significant difference on DHEA-S concentrations was observed between AD patients and healthy controls, when AD was not assessed by NINCDS-ARDRA or country of studies was not Italy. Interestingly, for subgroup analysis with high NOS scores, heterogeneity was notably lower compared with the heterogeneity of the overall analysis. To further investigate whether the severity forms of AD affected the overall outcome, two additional subgroup analyses, with respect to DHEA and DHEA-S, were performed. As regards DHEA, two exploratory subgroup analyses separating the different severity forms of AD were conducted, but there was no significant difference between individuals with different severity forms of AD and controls. Nevertheless, significantly lower DHEA-S was found in moderate AD patients compared to controls (k = 17, SMD = −1.12, 95% CI −1.71; −0.53, *Z* = −3.72, *p* = 0.002). Also, there was no significant difference between individuals with mild or severe AD and controls.

**Table 2:** DHEA subgroup analysis.

	**Number of studies**	**SMD (95% CI)**	***Z***	***P*-value**	**Heterogeneity**		
					**Q statistic (DF; *p*-value)**	**τ^2^**	***I*^2^**
All	15	0.51 [−0.44; 1.45]	1.06	0.291	307.02 14 < 0.0001	3.22	95.40%
**MATERIAL**							
CSF	4	2.13 [−1.49; 5.76]	1.15	0.249	156.50 3 < 0.0001	13.32	98.10%
Plasma	2	1.28 [−0.96; 3.52]	1.12	0.263	36.16 1 < 0.0001	2.55	97.20%
Serum	9	−0.22 [−1.02; 0.57]	−0.55	0.585	62.63 8 < 0.0001	1.23	87.20%
**STUDY COUNTRY**							
Other	5	−0.60 [−2.49; 1.30]	−0.62	0.537	144.40 4 < 0.0001	4.41	97.20%
USA	4	2.13 [−1.49; 5.76]	1.15	0.249	156.50 3 < 0.0001	13.32	98.10%
Sweden	6	0.40 [0.08; 0.72]	2.46	0.014	2.25 5 0.8132	0.00	0.00%
**MEAN AGE**							
>80	5	2.54 [0.32; 4.76]	2.24	0.025	166.00 4 < 0.0001	6.16	97.60%
≤ 80	10	−0.36 [−1.05; 0.34]	−1.01	0.313	61.74 9 < 0.0001	1.00	85.40%
**AD ASSESSMENT**							
NINCDS-ARDRA	12	0.64 [−0.55; 1.83]	1.05	0.292	287.93 11 < 0.0001	4.13	96.20%
Other	3	0.07 [−1.09; 1.24]	0.12	0.904	13.44 2 0.0012	0.87	85.10%
**COLLECTION TIME**							
AM	8	0.29 [−0.14; 0.72]	1.31	0.189	15.12 7 0.0345	0.20	53.70%
Other	7	0.72 [−1.27; 2.71]	0.70	0.481	276.30 6 < 0.0001	6.88	97.80%
**ASSAYED METHODS**							
RIA	9	0.07 [−0.33; 0.46]	0.34	0.737	17.23 8 0.0278	0.18	53.60%
Other	6	1.21 [−1.15; 3.57]	1.00	0.316	237.80 5 < 0.0001	8.34	97.90%
**SEVERITY OF AD**							
Moderate	11	0.04 [−0.83; 0.90]	0.08	0.937	150.10 10 < 0.0001	1.94	93.30%
Severe	4	2.13 [−1.49; 5.76]	1.15	0.249	156.50 3 < 0.0001	13.32	98.10%

*USA, United States of America; RIA, radioimmunoassay; CSF, cerebrospinal fluid; SMD, standardized mean difference; DF, degrees of Freedom; DSM, Diagnostic, and Statistical Manual of Mental Disorders; AD, Alzheimer's disease*.

**Table 3:** DHEA-S subgroup analysis.

	**Number of studies**	**SMD (95% CI)**	***Z***	***P*-value**	**Heterogeneity**		
					**Q statistic (DF; *p*-value)**	**τ^2^**	***I*^2^**
All	28	−0.69 [−1.17; −0.22]	−2.84	0.004	570.78 27 < 0.0001	1.49	95.30%
**MATERIAL**							
Plasma	11	−0.66 [−1.53; 0.21]	−1.49	0.136	185.75 10 < 0.0001	2.01	95.90%
Serum	17	−0.71 [−1.32; −0.11]	−2.31	0.021	376.20 16 < 0.0001	1.45	95.70%
**STUDY COUNTRY**							
Italy	7	−1.85 [−3.01; −0.69]	−3.12	0.002	150.94 6 < 0.0001	2.30	96.00%
Not Italy	21	−0.30 [−0.87; 0.27]	−1.05	0.296	411.53 20 < 0.0001	1.60	95.10%
**NOS**							
High	3	−0.12 [−0.47; 0.22]	−0.69	0.490	4.39 2 0.1116	0.05	54.40%
Medium	25	−0.74 [−1.32; −0.16]	−2.49	0.013	537.97 24 < 0.0001	2.02	95.50%
**AD ASSESSMENT**							
NINCDS-ARDRA	18	−0.68 [−1.27; −0.09]	−2.24	0.025	402.64 17 < 0.0001	1.48	95.80%
Other	10	−0.72 [−1.63; 0.20]	−1.54	0.125	166.36 9 < 0.0001	2.02	94.60%
**ASSAYED METHODS**							
RIA	17	−0.97 [−1.59; −0.35]	−3.06	0.002	333.42 16 < 0.0001	1.54	95.20%
Other	11	−0.23 [−1.14; 0.68]	−0.50	0.620	237.34 10 < 0.0001	2.16	95.80%
**SEVERITY OF AD**							
Mild	4	−0.33 [−1.98; 1.33]	−0.39	0.699	80.50 3 < 0.0001	2.72	96.30%
Moderate	17	−1.12 [−1.71; −0.53]	−3.72	0.002	361.47 16 < 0.0001	1.38	95.60%
Severe	7	0.23 [−1.07; 1.53]	0.35	0.729	123.91 6 < 0.0001	2.84	95.20%

*USA, United States of America; RIA, radioimmunoassay; SMD, standardized mean difference; DF, degrees of Freedom; DSM, Diagnostic, and Statistical Manual of Mental Disorders; AD, Alzheimer's disease*.

### Sensitivity and Bias Analysis

**Figure 4:**
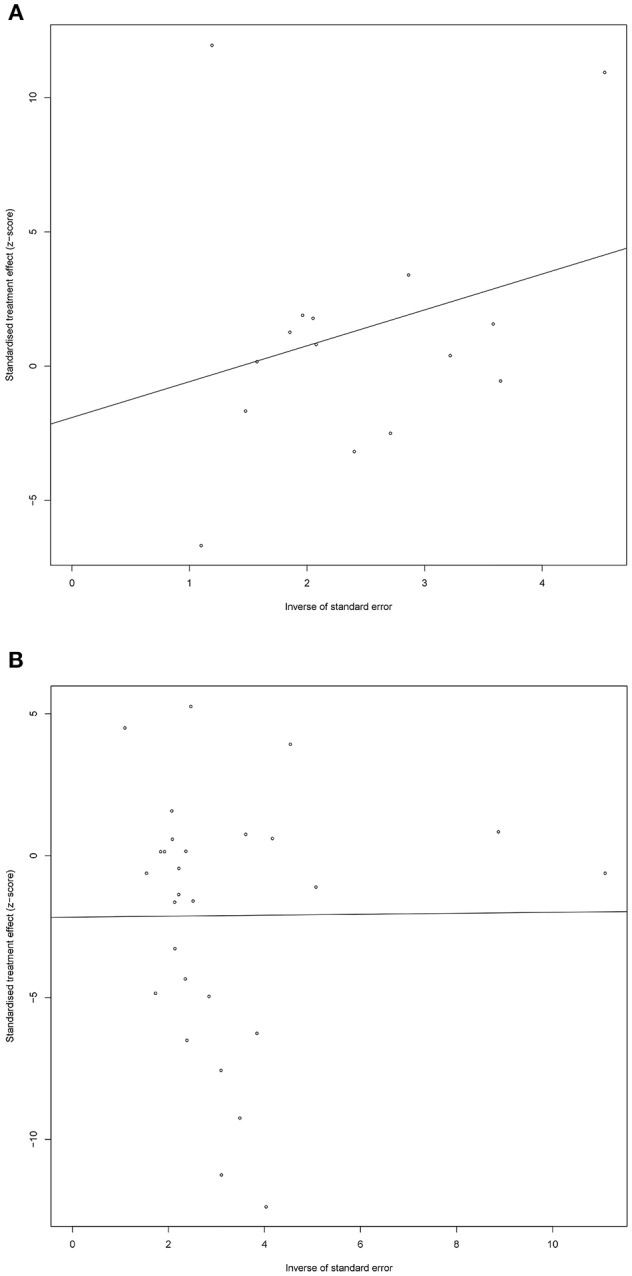
Egger funnel plots of DHEA **(A)** and DHEA-S **(B)**. Egger funnel plots to assess publication bias. Plots show study size as a function of effect size for studies included in the meta-analysis. The dots represent each study.

Sensitivity analysis showed little change in SMD and corresponding 95% CI after each individual study was omitted, one at a time, suggesting that the current meta-analysis data were relatively stable. The graphical Egger funnel plots of both DHEA and DHEA-S were symmetrical and Egger's tests scored *P*-values of 0.58 and 0.18 (Figure 4), respectively, confirming low possibility of publication bias of the included studies.

## Discussion

Our results showed that there was no association between AD and DHEA concentrations. On the other hand, DHEA-S concentrations were significantly lower in AD patients than in healthy controls. Several recent studies suggested that DHEA and DHEA-S levels are associated with AD, but the results are inconsistent. Several reviews have been published and they suggest that DHEA and DHEA-S levels may play a role in the risk of developing AD (Cho et al., 2006; Aldred and Mecocci, 2010). To the best of our knowledge, this is the first meta-analysis to explore the associations of DHEA and DHEA-S concentrations with AD status.

Noteworthy is the fact that DHEA is a testosterone precursor and a recent meta-analysis on testosterone concentrations and risk for AD found that lower plasma testosterone concentrations were significantly associated with increased risk for AD in the elderly subjects (Lv et al., 2016). Several lines of investigation have shown that testosterone in elderly subjects would improve the memory deficits of aging (Resnick et al., 2017). Moreover, a study proved that old male macaques have a reduced expression of enzyme 3β-hydroxysteroid dehydrogenase in the hippocampus (Sorwell et al., 2012). Consequently, they are less capable of centrally converting DHEA to testosterone, which may explain our finding of DHEA.

Regarding DHEA-S, the neuroactive steroids hypothesis of AD postulated that the changes in the brain, as a result of the disturbances of the processes involving the DHEA-S factors (Goncharov and Katsia, 2013), which indicated that DHEA-S has important roles in the changes in the brains of patients and cognitive function in the elderly men. Further investigations are necessary for better understanding of the molecular mechanisms that account for these changes of DHEA-S and DHEA concentrations in AD. Experimental research showed that DHEA-S is a major neurosteroid, which display beneficial neuroprotective properties and improve altered cognitive processes such as learning and memory (Klinge et al., 2018). Meanwhile, several studies reported that the reduction of steroid hormones during aging might play an important role in the onset and progression of neurodegenerative diseases (de Menezes et al., 2016). Concentrations of DHEA-S have been seen to decline with aging and this has been associated with immune system dysfunction, increased oxidative stress and cognitive decline (Klinge et al., 2018).

In addition, evidence has shown that DHEA-S also produces antioxidant and anti-inflammatory effects. Experimental studies have demonstrated that DHEA-S concentrations protect rat and human hippocampal neuronal cells against oxidative stress-induced cellular damage (Vieira-Marques et al., 2016; Nguyen et al., 2017). Of note, accumulating evidence indicates that proinflammatory cytokines are present in neuritic plaques (a hallmark of AD) and may be regulated by DHEA-S (Barger et al., 2000). Moreover, inflammation mediated by activated microglia is known as an important factor in AD's pathophysiology (Barger et al., 2000). Evidence has also shown that a supplement of DHEA-S greatly increases neuronal survival and differentiation and reduces astroglial proliferation rates in mouse brain cells in cultures (Bologa et al., 1987). Furthermore, existing data also suggested that treatment with 7β-Hydroxy-epiandrosterone (7β-OH-EpiA), a derivative credited with providing DHEA-S with its neuroprotective effect, prevents the Aβ-induced increase of Tau-protein immunoreactivity in rat hippocampus (Dudas et al., 2004).

Interestingly, the animal studies have shown that rats administered with DHEA-S have been proven to reduce the deficits in working, reference, and spatial memory (Maurice et al., 1998). These effects were found in several animal models: Aβ administration in the brain, with olfactory bulbectomy-induced cognitive impairment, senescence induced by D-galactose, and aging (Maurice et al., 1998; Markowski et al., 2001; Chen et al., 2008; Sakr et al., 2014). Clinical studies have shown that in the elderly, men can suffer cognitive deficits after interventions, such as androgen deprivation therapy for prostate cancer (McHugh et al., 2018). This intervention results in inactivation of the interactions between these hormones (testosterone, DHEA and DHEA-S) with target tissue or reducing their levels. As a result, there is a marked decrease of the effects that DHEA and DHEA-S have in organs and tissues (Sun et al., 2018). The preceding findings may provide valuable evidence about the causes of memory alterations in middle-aged and elderly subjects and androgen suppression. Thus, in AD, the dysfunction of DHEA-S has been hypothesized as a new player in pathophysiology of AD. Overall, this hypothesis is in line with our findings. Thus, DHEA-S may provide a basis for effective interventions to reduce AD in future.

Furthermore, subgroup analyses were performed in this study to see if results could change with different specified subgroups of the eligible studies. The specified subgroups included a relatively small number of studies and this compromised the statistical efficiency of the analyses. Thus, results of these subgroup analyses should be seen as exploratory and should be interpreted cautiously.

Contrary to results of the overall comparisons, this meta-analysis demonstrated that studies conducted in Sweden showed that DHEA concentrations were significantly higher in AD patients than in healthy controls. Our result may suggest that different countries or regions may form different homogenous groups when investigating the relationship between DHEA and AD. However, more future investigations are needed to ascertain this hypothesis. In this study, heterogeneity could not be evaluated in terms of social political economic, ethnicity, technical level, and cultural factors, because these characteristics were rarely reported in the eligible studies. Thus, we considered country of study as a likely substitute because it contains all those characteristics (Tafazzoli et al., 2018). In addition, this meta-analysis demonstrated that significantly higher DHEA concentrations were observed in AD patients than in healthy controls among studies which enrolled subjects aged 80 or older. There is a growing body of evidence suggesting that concentrations of DHEA and DHEA-S might decrease with age. It is also important to note that, in contrast with DHEA, DHEA-S concentrations were significantly lower in AD patients than in healthy controls. This would make sense because DHEA is a substrate for DHEA-S synthesis and, in this regard, it may be suggested that the activity of sulfatase or molecular mechanisms of DHEA-S bioconversion were impaired in patients with AD (Aly et al., 2011). Further investigations are necessary to understand the molecular mechanisms that account for the changes of DHEA and DHEA-S levels in AD patients. The decline in DHEA-S levels has been associated with different severity forms of AD (Valenti et al., 2009). Our findings suggest that the decline in DHEA-S levels may be a part of a decompensatory process in pathophysiology of moderate AD. This may explain why DHEA-S regulation was normal in the compensatory phase of mild AD, while the moderate AD developed to the decompensatory phase, which will result in different degrees of DHEA-S disorder. Moreover, the failure of this compensation mechanism may underlie the development of severe AD. Therefore, there is a clear need for more prospective studies to validate these hypotheses about the relation between the DHEA-S and different severity forms of AD. Also, this meta-analysis demonstrated no significant difference in the concentrations of DHEA-S between AD patients and healthy controls, when plasma DHEA-S was measured or assayed methods were not RIA. Our result may suggest that studies using serum sample and RIA when measuring the relationship between DHEA-S and AD may have higher homogeneity (Huayllas et al., 2018). However, considering that most of the eligible studies did not report the time lag between the sample collection and analysis, assay results might have been affected significantly. Also, these inherent variables in biochemical measurements, different assay methods and lack of a standardized method of assay, the wide time span over which different studies were conducted (in addition to other variables of study design), and study population, make the comparisons between different subgroups very unreliable (Tamae et al., 2013). It is inappropriate to deduce from subgroup analysis that the technology of RIA is more reliable than other technologies, or to deduce that serum sample is better for the analysis of DHEA-S than other samples. Therefore, more future investigations are needed to compare measurement methods and samples when studying the relationship between AD and DHEA-S. These investigations shall need to take into account all the confounding factors specified above in the research design.

### Limitations

This systematic review and meta-analysis is subject to several limitations. First, there was a limited number of eligible studies on the association between AD and concentrations of DHEA, leading to the lack of power in this analysis. Second, all studies for meta-analysis were case control in design, which could not make causality inference. Third, some factors that may influence concentrations of DHEA and DHEA-S, such as race/ethnicity, age, gender, body-mass index, smoking, drinking alcohol, and blood pressure were not examined due to limited sample size. High heterogeneity was observed for studies on both DHEA and DHEA-S concentrations between AD patients and healthy controls, which was not a surprise given the large variations in study designs and study populations (Villemagne and Chételat, 2016). Last, because we only focused on AD, MCI as a prodrome of AD was excluded (Ströhle et al., 2015). Thus, the analyses should be seen as exploratory and results should be interpreted cautiously.

## Conclusions

Despite the above limitations, the findings of this study have important implications. In particular, the results of this study support the hypothesis that lower DHEA-S concentrations may be related to the development of AD. Although whether DHEA-S could be used as a diagnostic tool requires further research. Prospective studies are needed to assess the mechanisms of long-term changes in DHEA and DHEA-S concentrations in the course of AD development. Also, large scale randomized controlled trials could demonstrate whether DHEA and DHEA-S supplementations could slow down cognitive decline in AD patients. The results of this type of randomized controlled trials could also provide evidence of the usefulness of DHEA and DHEA-S as biomarkers for AD.

## Author Contributions

XP and AL contributed to the study design, while XW and AK contributed to the data collection. Statistical analyses and interpretation of results were performed by XW, whereas AL and SW drafted the manuscript and edited the language. All the authors participated in the critical revisions, and approved the final version of the manuscript.

### Conflict of Interest Statement

The authors declare that the research was conducted in the absence of any commercial or financial relationships that could be construed as a potential conflict of interest.
