# Supplementary material for: Dehydroepiandrosterone and Dehydroepiandrosterone Sulfate in Alzheimer's Disease: A Systematic Review and Meta-Analysis
Source: Front Aging Neurosci. 2019 Mar 29;11:61. doi: 10.3389/fnagi.2019.00061 (PMC6449476; doi:10.3389/fnagi.2019.00061)
Supplement: Supplementary file 1 [file Table_1.DOC]

Search strategies: details of search strategy.

((((Alzheimer's Disease[Title] OR Dementia, Senile[Title] OR Senile Dementia[Title] OR Dementia, Primary Senile Degenerative[Title] OR Alzheimer Sclerosis[Title] OR Sclerosis, Alzheimer[Title] OR Alzheimer Syndrome[Title] OR Alzheimer Dementia[Title] OR Alzheimer Dementias[Title]))) AND ((androgen[Title/Abstract] OR neurosteroid[Title/Abstract] OR dehydroepiandrosterone[Title/Abstract] OR DHEA dehydroepiandrosteronesulfate[Title/Abstract] OR DHEA-S[Title/Abstract] OR Prasterone[Title/Abstract] OR Androstenolone[Title/Abstract] OR Sulfate, Dehydroepiandrosterone[Title/Abstract] OR Prasterone Sulfate[Title/Abstract] OR Sulfate, Prasterone[Title/Abstract] OR DHA Sulfate[Title/Abstract] OR Sulfate, DHA[Title/Abstract] OR Dehydroepiandrosterone Sulfate[Title/Abstract])))

111 of PubMed

(TITLE: ((((((((Alzheimer's Disease OR Dementia, Senile) OR Senile Dementia) OR AD) OR Alzheimer Sclerosis) OR Sclerosis, Alzheimer) OR Alzheimer Syndrome) OR Alzheimer Dementia) OR Alzheimer Dementias) AND TOPIC: ((((((((((((androgen OR neurosteroid) OR dehydroepiandrosterone) OR DHEA dehydroepiandrosteronsulfate) OR DHEA-S) OR Prasterone) OR androstanolone) OR Sulfate, Dehydroepiandrosterone) OR dhead) OR Sulfate, Prasterone) OR DHA Sulfate) OR Sulfate, DHA) OR Dehydroepiandrosterone Sulfate))

277 of Web of Science

('alzheimer(s)disease':ti OR 'dementia, senile':ti OR 'senile dementia':ti OR 'ad':ti OR 'alzheimer sclerosis':ti OR 'sclerosis, alzheimer':ti OR 'alzheimer syndrome':ti OR 'alzheimer dementia':ti OR 'alzheimer dementias':ti) AND ('androgen':ab,ti OR 'neurosteroid':ab,ti OR 'dehydroepiandrosterone':ab,ti OR 'dhea dehydroepiandrosteronesulfate':ab,ti OR 'dhea-s':ab,ti OR 'prasterone':ab,ti OR 'androstenolone':ab,ti OR 'sulfate, dehydroepiandrosterone':ab,ti OR 'dheas':ab,ti OR 'sulfate, prasterone':ab,ti OR 'dha sulfate':ab,ti OR 'sulfate, dha':ab,ti OR 'dehydroepiandrosterone sulfate':ab,ti)

167 of Embase

TX ( androgen OR neurosteroid OR dehydroepiandrosterone OR DHEA dehydroepiandrosteronesulfate OR DHEA-S OR Prasterone OR Androstenolone OR Sulfate, Dehydroepiandrosterone OR DHEAS OR Sulfate, Prasterone OR DHA Sulfate OR Sulfate, DHA OR Dehydroepiandrosterone Sulfate ) AND AB (Alzheimer's Disease OR Dementia, Senile OR Senile Dementia OR AD OR Alzheimer Sclerosis OR Sclerosis, Alzheimer OR Alzheimer Syndrome OR Alzheimer Dementia OR Alzheimer Dementias)

43 of PsycARTICLES
