# Supplementary material for: Dehydroepiandrosterone and Dehydroepiandrosterone Sulfate in Alzheimer's Disease: A Systematic Review and Meta-Analysis
Source: Front Aging Neurosci. 2019 Mar 29;11:61. doi: 10.3389/fnagi.2019.00061 (PMC6449476; doi:10.3389/fnagi.2019.00061)
Supplement: Supplementary file 2 [file Table_2.DOC]

**NEWCASTLE - OTTAWA QUALITY ASSESSMENT SCALE**

**🟑**This symbol represents the option and gets one point.

**Selection**

1) Is the case definition adequate?

a) yes, with independent validation **🟑**

b) yes, eg record linkage or based on self reports

c) no description

2) Representativeness of the cases

a) consecutive or obviously representative series of cases **🟑**

b) potential for selection biases or not stated

3) Selection of Controls

a) community controls **🟑**

b) hospital controls

c) no description

4) Definition of Controls

a) no history of disease (endpoint) **🟑**

b) no description of source

**Comparability**

5.6) Comparability of cases and controls on the basis of the design or analysis

a) study controls the most important factor. **🟑**

b) study controls for any additional factor **🟑**

**Outcome**

7) Ascertainment of exposure

a) secure record (eg surgical records) **🟑**

b) structured interview where blind to case/control status **🟑**

c) interview not blinded to case/control status

d) written self report or medical record only

e) no description

8) Same method of ascertainment for cases and controls

a) yes **🟑**

b) no

9) Non-Response rate

a) same rate for both groups **🟑**

b) non respondents described

c) rate different and no designation

**The NOS Score Scale**

| **Study** | **Score** | **Selection** | | | **Comparability** | | | | **Outcome** | | |
| --- | --- | --- | --- | --- | --- | --- | --- | --- | --- | --- | --- |
|  |  | **1** | **2** | **3** | **4** | **5** | **6** | **7** | | **8** | **9** |
| Aldred, 2010 | 5 | 1 | 0 | 0 | 1 | 1 | 0 | 1 | | 1 | 0 |
| Armanini et al., 2003 | 5 | 1 | 0 | 0 | 1 | 1 | 0 | 1 | | 1 | 0 |
| Attal-Khémis et al., 1998 | 6 | 1 | 0 | 1 | 1 | 1 | 0 | 1 | | 1 | 0 |
| Bo et al., 2006 | 8 | 1 | 1 | 1 | 1 | 1 | 1 | 1 | | 1 | 0 |
| Brown et al., 2003 | 5 | 1 | 0 | 0 | 1 | 1 | 0 | 1 | | 1 | 0 |
| Carlson et al., 1999 | 7 | 1 | 1 | 1 | 1 | 1 | 0 | 1 | | 1 | 0 |
| Cho et al., 2006 | 6 | 1 | 1 | 0 | 1 | 1 | 0 | 1 | | 1 | 0 |
| Ferrari et al., 2000 | 5 | 1 | 0 | 0 | 1 | 1 | 0 | 1 | | 1 | 0 |
| Gulnora et al., 2015 | 6 | 1 | 0 | 1 | 1 | 1 | 0 | 1 | | 1 | 0 |
| Hillen et al., 2000 | 8 | 1 | 1 | 1 | 1 | 1 | 1 | 1 | | 1 | 0 |
| Hoskin et al., 2004 | 6 | 1 | 1 | 0 | 1 | 1 | 0 | 1 | | 1 | 0 |
| Leblhuber et al., 1991 | 6 | 1 | 0 | 1 | 1 | 1 | 0 | 1 | | 1 | 0 |
| Leblhuber et al., 1992 | 5 | 1 | 0 | 0 | 1 | 1 | 0 | 1 | | 1 | 0 |
| Leblhuber et al., 1993 | 6 | 1 | 0 | 1 | 1 | 1 | 0 | 1 | | 1 | 0 |
| Magri et al., 2000 | 5 | 1 | 0 | 0 | 1 | 1 | 0 | 1 | | 1 | 0 |
| Marx et al., 2006 | 5 | 1 | 0 | 0 | 1 | 1 | 0 | 1 | | 1 | 0 |
| Masera et al., 2002 | 6 | 1 | 0 | 1 | 1 | 1 | 0 | 1 | | 1 | 0 |
| Murialdo et al., 2000 | 6 | 1 | 1 | 0 | 1 | 1 | 0 | 1 | | 1 | 0 |
| Naylor et al., 2007 | 5 | 1 | 0 | 0 | 1 | 1 | 0 | 1 | | 1 | 0 |
| Naylor et al., 2010 | 5 | 1 | 0 | 0 | 1 | 1 | 0 | 1 | | 1 | 0 |
| Nasman et al., 1991 | 5 | 1 | 0 | 0 | 1 | 1 | 0 | 1 | | 1 | 0 |
| Nasman et al., 1995 | 6 | 1 | 1 | 0 | 1 | 1 | 0 | 1 | | 1 | 0 |
| Nasman et al., 1996 | 8 | 1 | 1 | 1 | 1 | 1 | 1 | 1 | | 1 | 0 |
| Rasmuson et al., 1998 | 6 | 1 | 1 | 0 | 1 | 1 | 0 | 1 | | 1 | 0 |
| Rasmuson et al., 2002 | 6 | 1 | 0 | 1 | 1 | 1 | 0 | 1 | | 1 | 0 |
| Ray et al., 2013 | 5 | 1 | 0 | 0 | 1 | 1 | 0 | 1 | | 1 | 0 |
| Schneider et al., 1992 | 5 | 1 | 0 | 0 | 1 | 1 | 0 | 1 | | 1 | 0 |
| Schupf et al., 2006 | 5 | 1 | 0 | 0 | 1 | 1 | 0 | 1 | | 1 | 0 |
| Solerte et al., 1999 | 6 | 1 | 0 | 1 | 1 | 1 | 0 | 1 | | 1 | 0 |
| Sunderland et al., 1989 | 5 | 1 | 0 | 0 | 1 | 1 | 0 | 1 | | 1 | 0 |
| Yanase et al., 1996 | 5 | 1 | 0 | 0 | 1 | 1 | 0 | 1 | | 1 | 0 |
